# Helicopter vs. ground transport: outcomes of out-of-hospital cardiac arrest patients with intra-arrest transport

**DOI:** 10.1007/s00508-026-02747-3

**Published:** 2026-04-27

**Authors:** Florian Ettl, Matthias Pertl, Michael Poppe, Matthias Mueller, Ingrid Magnet, Christoph Schriefl, Luca Michael Kurz, Christian Schoergenhofer, Juergen Grafeneder

**Affiliations:** 1 Department of Emergency Medicine, Medical University of Vienna, Waehringer Guertel 18–20/6D, 1090 Vienna, Austria; 2 Department of Emergency Medicine, Hietzing Clinic, Vienna, Austria; 3 Department of Clinical Pharmacology, Medical University of Vienna, Vienna, Austria

**Keywords:** eCPR, Cardiopulmonary resuscitation, Air ambulances, Emergency medical services, Mechanical chest compression

## Abstract

**Objective:**

Deciding to transport a patient intra-arrest is difficult. The effect of the mode of transportation, either helicopter emergency medical services (HEMS) or ground emergency medical services (GEMS) and its potential link to neurological outcomes has not yet been assessed.

**Methods:**

We performed a retrospective cohort study using data from our Cardiac Arrest Registry. Adult out-of-hospital cardiac arrest (OHCA) patients transported intra-arrest were included. Neurological outcome at 6 months, measured using the cerebral performance category (CPC 1–2: good, CPC 3–5: poor), served as the primary endpoint. Associations between mode of transport and prehospital time with neurological outcome were examined using univariable and multivariable logistic regression.

**Results:**

We included 639 patients of whom 533 (83%) were transported by GEMS and 106 (17%) by HEMS. Patients transported by HEMS were younger (median age 54 years vs. 58 years) and more frequently had witnessed arrest and a shockable initial rhythm (55% vs. 42%). Median prehospital time did not differ significantly between groups (HEMS: 62 min, GEMS: 58 min). In univariable and multivariable analyses, the transport mode was not associated with a favorable neurological outcome at 6 months (*p* > 0.4). Neurological outcome was independently associated with arrest-related factors, including age, witnessed arrest, initial rhythm and cumulative adrenaline dose.

**Conclusion:**

For patients undergoing intra-arrest transport, the mode of transport (HEMS vs. GEMS) was not significantly associated with neurological outcomes at 6 months. Neurological results appeared to be more influenced by arrest-related factors than by the transport method in this cohort.

**Supplementary Information:**

The online version of this article (https://doi.org/10.1007/s00508-026-02747-3) contains supplementary material, which is available to authorized users.

## Introduction

Out-of-hospital cardiac arrest (OHCA) remains a leading cause of death worldwide, with overall survival and rates of favorable neurological outcomes remaining low despite significant advances in prehospital care [1]. Even under ideal conditions, most patients do not achieve return of spontaneous circulation (ROSC) at the scene [2]. For patients with promising cardiac arrest circumstances (e.g., witnessed arrest, bystander cardiopulmonary resuscitation CPR, shockable rhythm, high-quality advanced life support) [3], intra-arrest transport may enable access to physiology-guided CPR (with arterial line, transesophageal echocardiography [4]) and extracorporeal cardiopulmonary resuscitation (eCPR). This is supported by data showing that, in some cases, patients can still achieve good long-term outcomes [5]; however, transporting with ongoing chest compressions is logistically complex, resource-intensive (e.g., mechanical CPR device) [6] and may compromise the quality of CPR. Thus, its impact on patient outcomes remains uncertain [6]. Furthermore, it is unclear whether the mode of transport, i.e., helicopter emergency medical services (HEMS) versus ground emergency medical services (GEMS) influences outcomes. The benefits of HEMS transport include rapid transfer, especially over longer distances; however, the limited space inside the helicopter may hinder quick responses to issues such as inadvertent extubation or a slipping chest compression device. Moreover, helicopters are often unable to land near the patient, thereby negating the time advantage of a quick transfer. Current evidence supporting a benefit of HEMS over GEMS transport for intra-arrest transport is limited and most available studies focus on post-ROSC transport or on specific cardiac arrest groups (e.g., traumatic cardiac arrest) [7].

Therefore, this study aims to evaluate the influence of transportation mode (HEMS vs. GEMS) on neurological outcomes and survival rates among OHCA patients with intra-arrest transport to the hospital.

## Methods

### Study design and setting

This is a retrospective data analysis conducted at a single center, approved by the Ethics Committee of the Medical University of Vienna (Ethics Committee Number: 2376/2020). The research was conducted within the Department of Emergency Medicine at the Medical University of Vienna, Vienna General Hospital. The Vienna General Hospital functions as a tertiary care university hospital. The Department of Emergency Medicine serves more than 60,000 inpatients annually and is a certified cardiac arrest and high-volume eCPR center, treating about 250–300 patients in cardiac arrest and performing 60–70 eCPR treatments annually [8]. Vienna, a city with 2 million inhabitants, is covered by a municipal GEMS program with paramedics trained in advanced life support, including airway management, intravenous access and drug administration. Prehospital physicians are dispatched to every cardiac arrest. During the study period, 7–9 GEMS units staffed with emergency physicians operated within the city of Vienna. Additionally, one HEMS unit was based in Vienna, with two more nearby, collectively serving the metropolitan area (2 million inhabitants) and surrounding regions (additionally approximately 1 million inhabitants). The decision to dispatch GEMS or HEMS was made at the dispatcher’s discretion or upon request from the GEMS team on site (alerting a HEMS unit if a quicker patient transport is expected). During the study period, no standardized institutional eCPR eligibility criteria were formally implemented within our emergency medical system. Structured eCPR selection criteria, including defined time thresholds to hospital arrival, were introduced only later [9]. Both HEMS and GEMS were equipped with mechanical chest compression devices (Corpuls CPR, GS Elektromedizinische Geräte G. Stemple GmbH, Kafering, Germany; LUCAS 2 or 3, Jolife AB/Physio Control/Stryker, Lund, Sweden; Autopulse, Zoll Medical Corporation, Chelmsford, MA, USA).

### Data collection

The Cardiac Arrest Registry of the Department of Emergency Medicine (Ethics Committee Number: 1219/2018) served as the source for this retrospective analysis. It records all patients treated for cardiac arrest at our clinic. Neurological outcomes were evaluated by a trained team during in-hospital care through direct patient assessments and after discharge via structured telephone interviews with patients or, when necessary, their caregivers, using a standardized questionnaire. Because of the retrospective study design, assessors were not formally blinded to the transport modality. Further details on the collected data and follow-up have been previously published [10].

### Inclusion criteria

Adult patients (≥ 18 years) admitted to our department with non-traumatic OHCA and intra-arrest transport between January 2013 and May 2021 by GEMS or HEMS.

### Exclusion criteria

Excluded were in-hospital cardiac arrests, cases without ongoing CPR upon arrival at our clinic and cases with incomplete data.

### Objectives

The primary objective was to assess the association between the mode of transportation (HEMS vs. GEMS) and a favorable neurological outcome at 6 months in patients with OHCA and intra-arrest transport.

Secondary objectives included: (i) the association between prehospital time and neurological outcome at 6 months and (ii) the association of mode of transport and neurological outcome at 6 months after correction for established arrest-related predictors of neurological outcome at 6 months. Prehospital time was defined as the interval from the initial EMS call to hospital arrival, including dispatch, potential secondary HEMS activation, on-scene resuscitation, and transport.

### Primary endpoint

The primary endpoint was a favorable neurological outcome at 6 months. A favorable neurological outcome was defined as a cerebral performance category (CPC) score of 1 or 2.

### Statistical methods

Patients were divided into groups based on mode of transport, either GEMS or HEMS. Baseline characteristics were summarized using counts and percentages for categorical data and means with standard deviations or medians with interquartile ranges for continuous data, as appropriate. Differences between transport groups were assessed using Fisher’s exact test for categorical variables and Student’s t‑test or the Mann-Whitney U test for continuous variables.

The association between transport mode and neurological outcomes at 6 months (defined as a CPC score of 1–2 for favorable neurological outcomes vs. CPC 3–5 for unfavorable neurological outcomes or death) was analyzed using univariate and multivariable logistic regression. The transport mode served as the main independent variable. Based on clinical reasoning and previous publications [3] we made adjustments for age (in years), sex (male/female), witnessed arrest (yes/no), bystander CPR (yes/no), initial cardiac rhythm (shockable vs. nonshockable) and total adrenaline dose (in mg). As a sensitivity analysis, we included prehospital time in the model described. To evaluate potential temporal bias, we analyzed the distribution of transport modes (HEMS vs. GEMS) over the study period using a χ^2^-test and logistic regression to identify any linear temporal trends. All statistical tests were two-sided, and *p*-values < 0.05 were considered statistically significant.

## Results

**Fig. 1:**
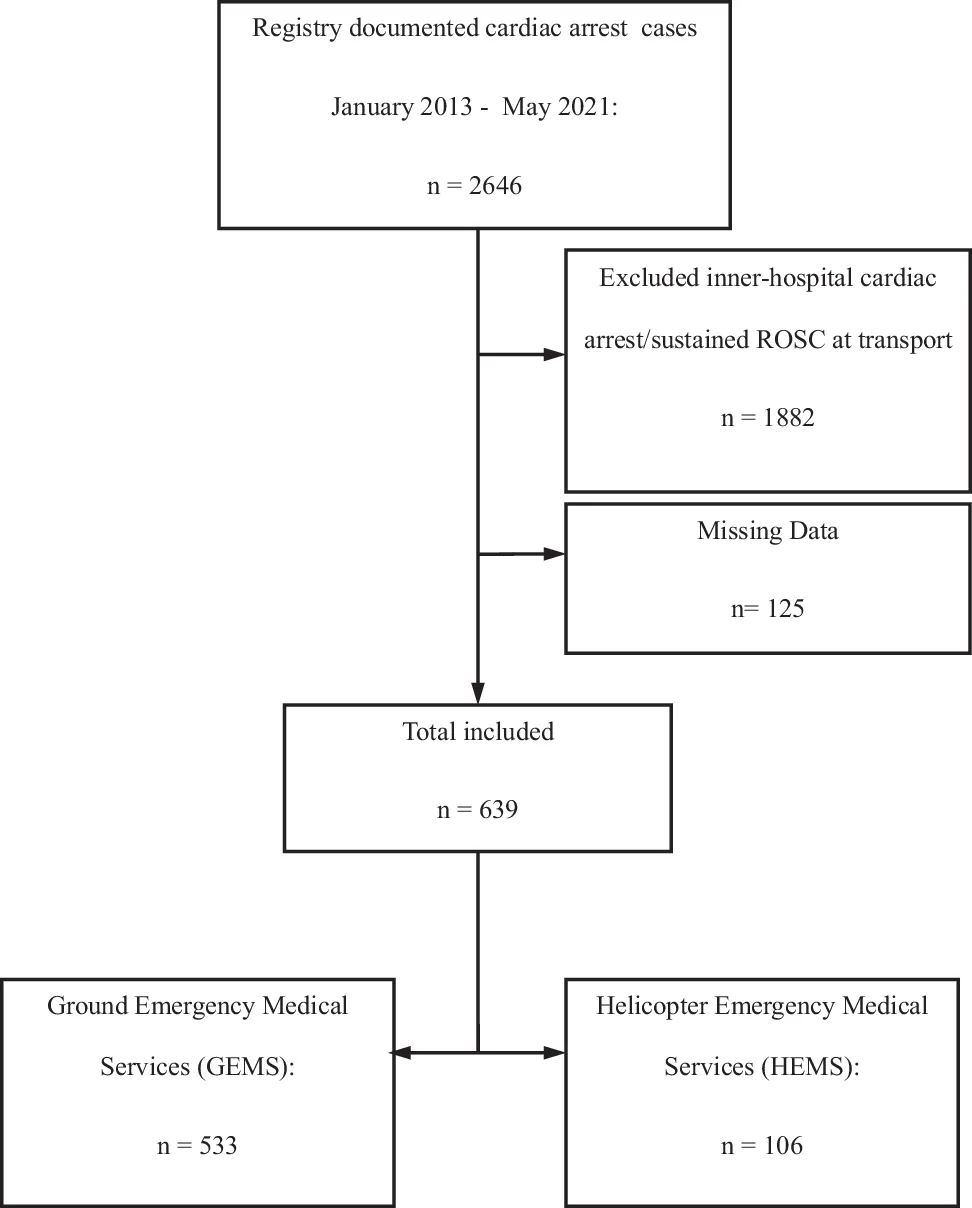
Flow diagram of patient inclusion and exclusion. Adult patients from our department’s Cardiac Arrest Registry (January 2013 to May 2021) were screened for eligibility. Patients were excluded based on predefined criteria, including in-hospital cardiac arrest, sustained return of spontaneous circulation (ROSC) before transport, and missing data. The final study cohort comprised patients undergoing intra-arrest transport and was divided by transport modality: helicopter emergency medical services (HEMS) and ground emergency medical services (GEMS)

We recorded 2646 OHCA cases between January 2013 and May 2021. After applying exclusion criteria, 639 patients were included in the final analysis: 533 (83%) transported via GEMS and 106 (17%) via HEMS (Fig. 1). The proportion of patients transported by HEMS remained stable over time (Figure S1, Table S1) with no significant variation across years (*p* = 0.869) and no significant temporal trend (odds ratio per year, 0.94; 95% confidence interval, CI, 0.86–1.03; *P* = 0.190). Among patients who underwent eCPR, 26% (*n* = 36) were transported by HEMS and 74% (*n* = 103) by GEMS. The proportion of eCPR patients transported by HEMS remained stable over time (Table S2) with no significant variation across years (*p* = 0.978) and no significant temporal trend (odds ratio per year, 0.93; 95% CI, 0.80–1.09; *P* = 0.369).

Baseline characteristics of the study population grouped by transportation are shown in Table 1. The median age of patients transported by HEMS was 4 years younger than that of patients transported by GEMS (*p* = 0.017). The body mass index (BMI) showed no significant differences between the transport groups (*p* = 0.4).

**Table 1:** Baseline characteristics

Characteristic	Overall *N* = 639^1^	GEMS *N* = 533^1^	HEMS *N* = 106^1^
Age (years), median (IQR)	57.0 (47.0–68.0)	58.0 (48.0–68.0)	54.0 (43.0–65.0)
Female, *n* (%)	140 (22%)	120 (23%)	20 (19%)
Body mass Index, median (IQR)	27.6 (24.7–30.9)	27.7 (24.7–30.9)	27.5 (24.7–29.4)
Witnessed arrest, *n *(%)	503 (79%)	426 (80%)	77 (73%)
Bystander CPR, *n *(%)	461 (72%)	373 (70%)	88 (83%)
Shockable rhythm, *n *(%)	281 (44%)	223 (42%)	58 (55%)
Prehospital time (min), median (IQR)	59.0 (48.6–71.0)	58.0 (48.0–71.0)	61.5 (52.0–73.0)
Cumulative adrenaline (mg), median (IQR)	8.0 (6.0–11.0)	8.0 (6.0–11.0)	7.5 (5.0–10.0)
pH on admission^2^, median (IQR)	6.9 (6.8–7.1)	6.9 (6.8–7.1)	6.9 (6.7–7.1)
Lactate on admission (mmol/L)^2^, median (IQR)	12.5 (9.6–16.0)	12.7 (9.8–16.0)	11.4 (8.0–15.0)
30-day mortality, *n *(%)	592 (93%)	497 (93%)	95 (90%)
6‑month mortality, *n *(%)	598 (94%)	501 (94%)	97 (92%)
30-day CPC 1 or 2, *n *(%)	28 (4.4%)	20 (3.8%)	8 (7.5%)
6‑month CPC 1 or 2, *n *(%)	32 (5.0%)	24 (4.5%)	8 (7.5%)

*IQR* interquartile range; *HEMS* helicopter emergency medical services; *GEMS* ground emergency medical services; *CPR* cardiopulmonary resuscitation; *CPC* cerebral performance category

^1^ Median (Q1–Q3); *n* (%) ^2^ obtained by blood gas analysis

The proportion of witnessed collapses was higher in the GEMS group than in the HEMS group, although this difference did not reach statistical significance (*p* = 0.12). The rates of bystander CPR and the proportion of patients presenting with a shockable initial rhythm were significantly higher in the HEMS group (bystander CPR: *p* = 0.006; shockable initial rhythm: *p* = 0.018). Prehospital time did not differ significantly between transportation modes (*p* = 0.2). The total adrenaline dose did not differ significantly between the groups (*p* = 0.2). Similarly, there were no significant differences in parameters obtained by the blood gas analysis on admission, including pH (*p* = 0.5) and lactate (*p* = 0.2).

**Fig. 2:**
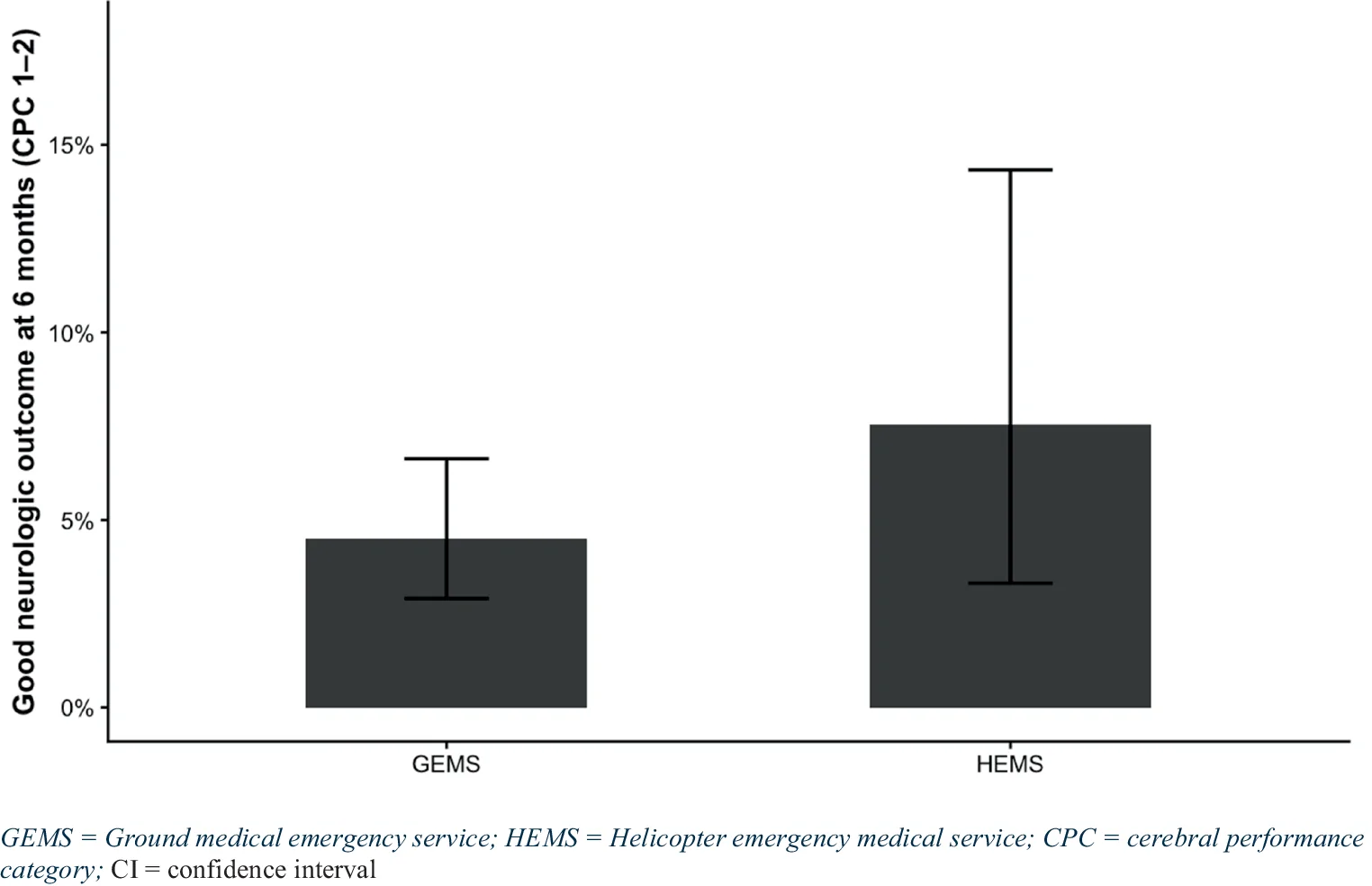
Rate of favorable neurologic outcome (CPC 1–2) at 6 months according to mode of transport with 95% CI. *GEMS* ground medical emergency service; *HEMS* helicopter emergency medical service; *CPC* cerebral performance category; *CI* confidence interval

**Fig. 3:**
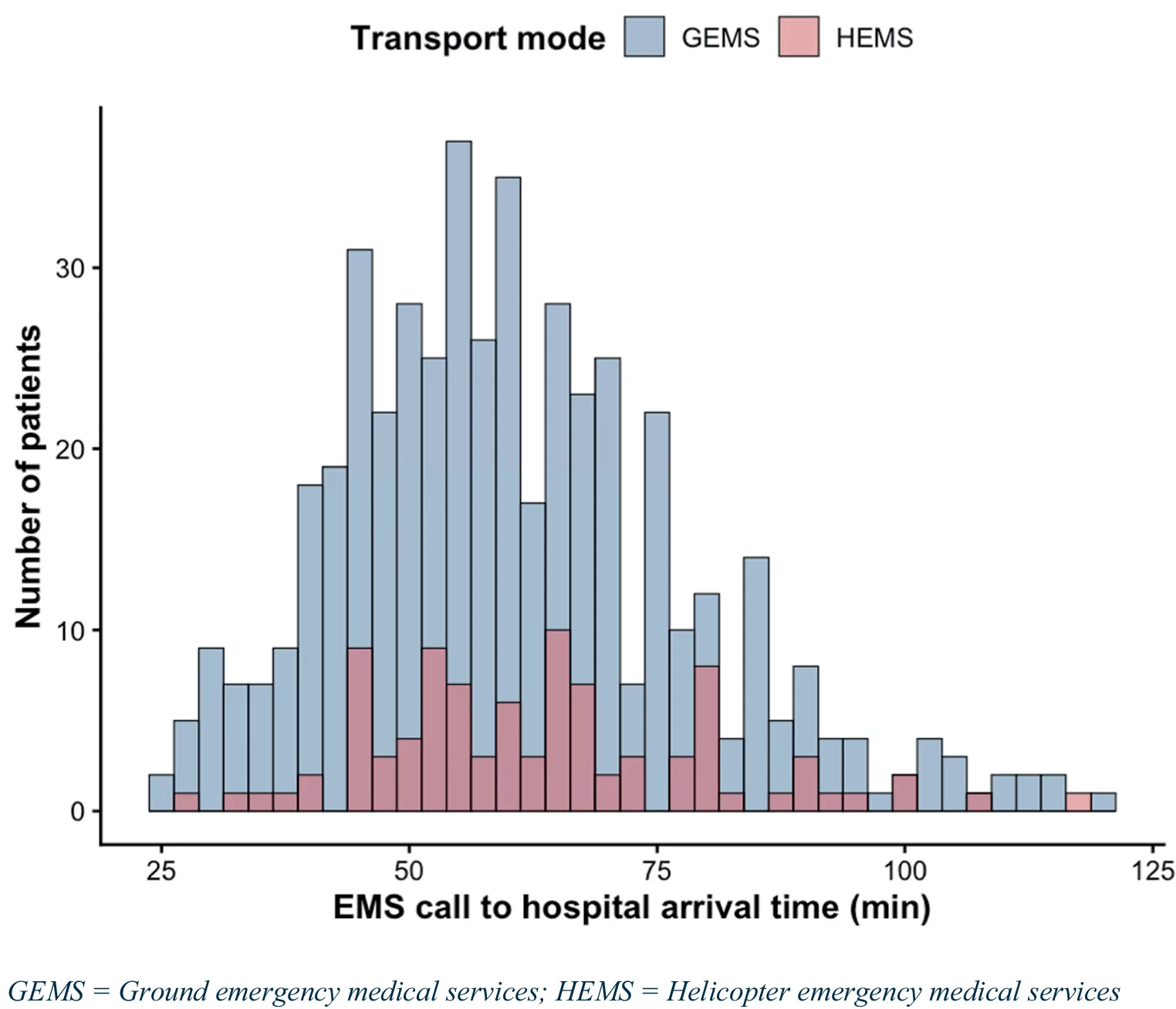
Distribution of prehospital time by transport mode. *GEMS* ground emergency medical services; *HEMS* helicopter emergency medical services

The mode of transportation was not significantly associated with a favorable neurologic outcome at 6 months (crude odds ratio OR 1.73; 95% CI: 0.71–3.81, *p* = 0.19; Table 2 and visualization Fig. 2). Prehospital time was also not associated with neurological outcome (crude OR 0.99; 95% CI, 0.97–1.01; *p* = 0.50; Fig. 3). The distribution of prehospital time by transport mode is shown in Fig. 3. In a multivariable logistic regression analysis, transport mode did not demonstrate an independent association with neurological outcomes at 6 months (adjusted OR, 1.52; 95% CI, 0.58–3.67; *p* = 0.37). This did not change when prehospital time was added (*n* = 540; adjusted OR for transport mode: 1.47; 95% CI, 0.54–3.74; *p* = 0.43; adjusted OR for transport duration per minute: 1.01; 95% CI, 0.98–1.03; *p* = 0.65; Supplementary Table S3). Factors established in OHCA-related contexts exhibited strong associations with neurological outcomes (see Table 2).

**Table 2:** Adjusted odds ratios (OR) with 95% confidence intervals (CI) of resuscitation characteristics and favorable neurologic outcome (CPC 1–2) at 6 months

Variable	Crude OR (95% CI)	*p*	Adjusted OR (95% CI)	*p*
HEMS transport (vs. GEMS)	1.73 (0.71–3.81)	0.19	1.52 (0.58–3.67)	0.370
Age (years)	–	–	0.97 (0.94–1)	0.028
Male sex	–	–	1.35 (0.48–4.85)	0.600
Witnessed arrest	–	–	12.94 (2.4–245.87)	0.017
Bystander CPR	–	–	2.54 (0.91–9.1)	0.100
Shockable first rhythm	–	–	21.65 (6.23–137.37)	< 0.001
Cumulative adrenaline (mg)	–	–	0.81 (0.72–0.91)	< 0.001

*HEMS* helicopter emergency medical services; *GEMS* ground emergency medical services; *CPR* cardiopulmonary resuscitation

## Discussion

This retrospective analysis of OHCA patients undergoing intra-arrest transport found that the mode of transport (HEMS vs. GEMS) was not significantly associated with 6‑month neurological outcomes, even after adjusting for established prognostic factors. Similarly, prehospital time was not significantly associated with favorable neurological recovery. Instead, outcomes appeared more closely linked to arrest-specific factors such as witnessed arrest, initial shockable rhythm and the total adrenaline dose, which indicates resuscitation duration. These findings suggest that within this selected group, resuscitation circumstances may have a greater impact on neurological outcomes than the mode of transport. Notably, the distribution of HEMS and GEMS transports remained consistent over the study period, reducing the likelihood of temporal bias.

Previous studies on HEMS use in OHCA have reported mixed results, likely reflecting heterogeneous patient populations and differences in study design [11]. In contrast, our cohort was limited to patients undergoing intra-arrest transport with ongoing CPR at hospital admission, thereby improving comparability but potentially limiting generalizability. While most prior studies have focused on short-term outcomes [12, 13], this is the first to examine 6‑month outcomes in this cohort. Long-term neurological recovery is increasingly recognized as the most clinically relevant endpoint, particularly in the context of eCPR [10].

In this cohort, there was no significant association between transport mode or prehospital time and a favorable neurological outcome at 6 months; however, due to the low event rates and the small number of patients receiving eCPR, these findings should be interpreted cautiously and should not be seen as evidence that reducing time to definitive treatment is unimportant.

The observed associations between arrest-specific factors and outcome are biologically plausible. The prognosis after cardiac arrest is mostly determined early in resuscitation, with factors like witnessed collapse, bystander CPR and shockable rhythm indicating short no-flow time and preserved organ function [3, 14, 15]. The association between cumulative adrenaline dose and outcome probably reflects resuscitation length rather than an independent effect. Once prolonged no-flow or low-flow states occur, neither transport method nor speed can likely reverse the established hypoxic-ischemic injury. Therefore, differences in transport logistics may have a limited impact on neurological outcomes in this group.

From a systems-of-care perspective, our findings suggest that HEMS does not consistently offer a survival benefit over GEMS during intra-arrest transport; however, the absence of a distinct advantage does not imply harm nor demonstrate their equivalence. Our results suggest that the challenges associated with HEMS transport do not negatively impact patient outcomes. This reinforces the idea that HEMS can enhance access to specialized treatment such as eCPR by helping patients in remote areas reach suitable centers. Therefore, HEMS can primarily increase access to advanced care rather than directly improving outcomes. Our findings do not indicate that the limitations inherent in air transport, such as confined space or potentially restricted treatment options, negatively affect patient outcomes. Decisions regarding intra-arrest transport should consider factors such as access to specialized resources [16, 17], safety and system efficiency [8, 18], while emphasizing early resuscitation efforts, which remain crucial [19–21].

We chose not to include geographic distance in our calculations. When assessing transport strategies for OHCA, relying solely on geographic distance does not provide a complete measure of clinical importance. From a prehospital and systems-of-care perspective, the critical factor is how quickly a patient can reach definitive in-hospital treatment after activation of the emergency system [10, 16]. This principle applies equally to GEMS and HEMS transport. Specifically, for HEMS, straight-line or road distances do not account for essential operational factors, such as aircraft activation time, available landing sites, secondary ground transfers between the landing zone and the patient or hospital, and the time needed for patient packaging and loading. Prior studies have shown that helicopter mission ground time, which includes these processes, can vary widely, typically ranging from 10–30 min [22]. Another study showed that the transportation time for patients with ST-segment elevation myocardial infarction (STEMI) transferred 30 miles for primary percutaneous coronary intervention (PCI) was shorter when ground transport was used instead of air transport [23]. Therefore, we focused on the interval from emergency call to hospital admission as a clinically relevant time-based metric that incorporates these real-world operational factors and more accurately reflects the decision-making process used in emergency medical services.

## Limitations

Several limitations should be considered. The retrospective design and reliance on registry data introduce potential biases, including selection bias, misclassification and unmeasured confounding. Transport modality was not randomly assigned but was influenced by clinical judgment and system-level factors, such as resource availability, transport logistics and termination of resuscitation decisions, which were not guided by standardized eCPR criteria during the study period. These factors may have impacted patient selection and limited the ability to draw causal conclusions from the findings.

The single center design and the urban setting, characterized by relatively short transport distances as well as a physician-staffed emergency medical system, may further limit the generalizability of our results to other regions with different organizational structures. In this context, the potential advantages of HEMS may have been less pronounced and our findings may not be directly applicable to rural or geographically remote settings. Our study may be underpowered to detect small but clinically relevant differences between transport modalities. The limited number of favorable neurological outcomes led to wide confidence intervals and post hoc power analyses confirmed limited statistical power across various endpoints (Supplement Table S3). Accordingly, the absence of statistically significant associations should be interpreted as a lack of demonstrated benefit rather than proof of equivalence.

Detailed prehospital time intervals (e.g., response, on-scene and transport times) were not consistently available and could not be reliably reconstructed. Therefore, total prehospital time from EMS call to hospital arrival was used as the most robust and comparable time variable.

Data on the modality of chest compressions during transport were incomplete. Although mechanical CPR was routinely used, detailed information on the use of mechanical versus manual CPR was not consistently available, particularly in the GEMS group and may represent a potential confounder.

Advanced airway management variables were not included in the multivariable analysis due to limited variability as nearly all patients were correctly intubated and mechanically ventilated prior to hospital arrival. This may not apply to other settings where airway management approaches differ and could affect resuscitation quality and outcomes.

Finally, neurological outcome was assessed using the cerebral performance category scale, which, although widely used, provides only a limited measure of functional recovery.

## Conclusion

Among OHCA patients undergoing intra-arrest transport to the hospital, neither the mode of transport nor prehospital time was associated with neurological outcomes at 6 months in this cohort. Neurological recovery mainly seemed to depend on early arrest features. These results emphasize the importance of early resuscitative efforts and appropriate patient selection for advanced (post)resuscitation treatment.; however, due to limited statistical power, small differences between transport strategies cannot be ruled out.

## Supplementary Information

ESM1: Supplementary material 1

## Data Availability

The datasets generated during the current study is available from the corresponding author on reasonable request.
