# Supplementary material for: Helicopter vs. ground transport: outcomes of out-of-hospital cardiac arrest patients with intra-arrest transport
Source: Wien Klin Wochenschr. 2026 Apr 27;138(21-22):665–72. doi: 10.1007/s00508-026-02747-3 (PMC13759648; doi:10.1007/s00508-026-02747-3)
Supplement: Supplementary file 1 [file 508_2026_2747_MOESM1_ESM.docx]

HEMS Supplementary Material

**Table S1.** Distribution of transportation mode by study year. Values represent the number (percentage) of patients transported via ground emergency medical services (GEMS) or helicopter emergency medical services (HEMS).

| **Year** | **Total** | **GEMS n (%)** | **HEMS n (%)** |
| --- | --- | --- | --- |
| 2013 | 61 | 49 (80.3%) | 12 (19.7%) |
| 2014 | 59 | 48 (81.4%) | 11 (18.6%) |
| 2015 | 66 | 55 (83.3%) | 11 (16.7%) |
| 2016 | 60 | 49 (81.7%) | 11 (18.3%) |
| 2017 | 84 | 66 (78.6%) | 18 (21.4%) |
| 2018 | 106 | 92 (86.8%) | 14 (13.2%) |
| 2019 | 77 | 66 (85.7%) | 11 (14.3%) |
| 2020 | 95 | 81 (85.3%) | 14 (14.7%) |
| 2021* | 31 | 27 (87.1%) | 4 (12.9%) |

*Inclusion until the end of May;

**Table S2.** Distribution of Patients Undergoing eCPR According to Transport Mode.

| **Year** | **Total eCPR** | **HEMS** | **GEMS** |
| --- | --- | --- | --- |
| Overall | 139 | 36/139 (25.9%) | 103/139 (74.1%) |
| 2013 | 15 | 4/15 (26.7%) | 11/15 (73.3%) |
| 2014 | 9 | 3/9 (33.3%) | 6/9 (66.7%) |
| 2015 | 10 | 4/10 (40.0%) | 6/10 (60.0%) |
| 2016 | 11 | 3/11 (27.3%) | 8/11 (72.7%) |
| 2017 | 13 | 4/13 (30.8%) | 9/13 (69.2%) |
| 2018 | 28 | 6/28 (21.4%) | 22/28 (78.6%) |
| 2019 | 18 | 4/18 (22.2%) | 14/18 (77.8%) |
| 2020 | 27 | 6/27 (22.2%) | 21/27 (77.8%) |
| 2021* | 8 | 2/8 (25.0%) | 6/8 (75.0%) |

*Inclusion until the end of May; eCPR… extracorporeal cardiopulmonary resuscitation; HEMS… helicopter emergency services; GEMS … ground emergency medical services.

**Table S3**. Adjusted Odds Ratios (OR) with 95% Confidence Interval (CI), of resuscitation characteristics and favorable neurologic Outcome (CPC1-2) at 6 months, only for patients with available prehospital time.

| **Variable** | **Adjusted OR (95% CI)** | **p-value** |
| --- | --- | --- |
| Age, per year | 0.96 (0.93–0.99) | 0.017 |
| Male sex | 1.37 (0.47–5) | 0.590 |
| Witnessed arrest | 10.94 (2.11–202.21) | 0.023 |
| Bystander CPR | 2.66 (0.92–9.76) | 0.096 |
| Shockable first rhythm | 47.3 (9.49–863.84) | < 0.001 |
| Cumulative adrenaline, per mg | 0.8 (0.7–0.91) | <0.001 |
| HEMS transport (vs GEMS) | 1.47 (0.54–3.74) | 0.430 |
| Transport time, per min | 1.01 (0.98–1.03) | 0.650 |

**Table S4.** Post hoc power calculation for ground emergency medical services (GEMS, n=533) or helicopter emergency medical services (HEMS, n=106). CPC … Cerebral Performance Category)

| **Outcome** | **GEMS rate** | **HEMS rate** | **Power** |
| --- | --- | --- | --- |
| CPC 1 or 2 at 6 months | 4.5 | 7.5 | 0.23 |
| CPC 1 or 2 at 1 month | 3.8 | 7.5 | 0.35 |
| CPC 1 or 2 at 1 year | 3.4 | 2.8 | 0.06 |
| 30-day mortality | 93.2 | 89.6 | 0.23 |

A)

B)

**Figure S1.** Distribution of transport modes by study year. Bar charts show the proportions of patients transported to the hospital by ground emergency medical services (GEMS) and helicopter emergency medical services (HEMS) intra-arrest for out-of-hospital cardiac arrest. Each bar represents one study year and totals 100% of transports for that year (A) or displays the absolute numbers (B). 2021 was included until the end of May.
